# The effectiveness of heat prevention plans in reducing heat-related mortality across Europe

**DOI:** 10.1088/1748-9326/ae2775

**Published:** 2025-12-23

**Authors:** Aleš Urban, Veronika Huber, Salomé Henry, Nuria Pilar Plaza, Lucie Tušlová, Shouro Dasgupta, Pierre Masselot, Ivana Cvijanovic, Malcolm Mistry, Mathilde Pascal, Francesca de’Donato, Claudia Di Napoli, Simon N Gosling, Silvia Kohnová, Jan Kyselý, Samuel Lüthi, Louis-François Pau, Martina S Ragettli, Reija Ruuhela, Niilo Ryti, Susana Das Neves Pereira da Silva, Shiri Zemah-Shamir, Wim Thiery, Ana-Maria Vicedo-Cabrera, Joanna Wieczorek, Francesco Sera, Ben Armstrong, Antonio Gasparrini, On behalf of the PROCLIAS TG 3.11, the MCC Collaborative Research Network

**Affiliations:** 1 Faculty of Environmental Sciences, Czech University of Life Sciences Prague, Prague, Czech Republic; 2 Institute of Atmospheric Physics, Czech Academy of Sciences, Prague, Czech Republic; 3 Doñana Biological Station (EBD), Spanish National Research Council (CSIC), Sevilla, Spain; 4 Institute of Epidemiology, Helmholtz Zentrum München—German Research Center for Environmental Health (GmbH), Neuherberg, Germany; 5 AgroParisTech, Paris, France; 6 Centro de Investigaciones sobre Desertificación, Consejo Superior de Investigaciones Científicas (CIDE, CSIC-UV-Generalitat Valenciana), Climate, Atmosphere and Ocean Laboratory (Climatoc-Lab), Moncada, Valencia, Spain; 7 Centro Euro-Mediterraneo sui Cambiamenti Climatici (CMCC), Venezia, Italy; 8 Environment & Health Modelling (EHM) Lab, Department of Public Health Environments and Society, London School of Hygiene & Tropical Medicine, London, United Kingdom; 9 ESPACE-DEV, Univ Montpellier, IRD, Montpellier, France; 10 Department of Economics, Ca’ Foscari University of Venice, Venice, Italy; 11 Santé Publique France, Department of Environmental and Occupational Health, French National Public Health Agency, Saint Maurice, France; 12 Department of Epidemiology, Lazio Regional Health Service, ASL ROMA 1, Rome, Italy; 13 European Centre for Medium-Range Weather Forecasts, Reading, United Kingdom; 14 School of Geography, University of Nottingham, Nottingham, United Kingdom; 15 Department of Land and Water Resources Management, Faculty of Civil Engineering, Slovak University of Technology, Bratislava, Slovakia; 16 Weather and Climate Risks Group, Institute for Environmental Decisions, ETH Zürich, Switzerland; 17 Rotterdam School of Management, Erasmus University Rotterdam, Rotterdam, The Netherlands; 18 Copenhagen Business School, Copenhagen, Denmark; 19 Upgötva AB, Stockholm, Sweden; 20 Swiss Tropical and Public Health Institute, Allschwill, Switzerland; 21 University of Basel, Basel, Switzerland; 22 Weather and Climate Change Impact Research, Finnish Meteorological Institute, Helsinki, Finland; 23 Center for Environmental and Respiratory Health Research (CERH), Research Unit of Population Health, University of Oulu, Oulu, Finland; 24 Department of Public Health, University of Helsinki, Helsinki, Finland; 25 Department of Epidemiology, Instituto Nacional de Saúde Dr Ricardo Jorge, Lisbon, Portugal; 26 School of Sustainability, Reichman University, Herzliya, Israel; 27 Department of Water and Climate, Vrije Universiteit Brussel, Brussels, Belgium; 28 Institute of Social and Preventive Medicine, University of Bern, Bern, Switzerland; 29 Oeschger Centre for Climate Change Research, University of Bern, Bern, Switzerland; 30 Centre of Numerical Weather Prediction, Institute of Meteorology and Water Management—National Research Institute, Warsaw, Poland; 31 Department of Statistics, Computer Science and Applications G. Parenti, University of Florence, Florence, Italy

**Keywords:** heat prevention plans, heat and health warning systems, heat-related mortality, heat adaptation

## Abstract

Heat-health warning systems and action plans, referred to as heat prevention plans (HPPs), are key public health interventions aimed at reducing heat-related mortality. Despite their importance, prior assessments of their effectiveness have yielded inconsistent results. The objective of this study is to systematically assess the effectiveness of HPPs in reducing heat-related mortality risk across Europe. We analysed daily mortality and mean temperature data from 102 locations in 14 European countries between 1990 and 2019. Using data from national experts, we identified the year of HPP implementation and categorised their development class. A three-stage analysis was conducted: (1) quasi-Poisson time series models were used to estimate location-specific warm-season exposure-response functions in 3 year subperiods; (2) mixed-effect meta-regression models with multilevel longitudinal structures were employed to quantify changes in pooled exposure-response functions due to HPP implementation, adjusted for long-term trends in heat-related mortality risks; and (3) the heat-related excess mortality due to HPP was calculated by comparing factual (with HPP) and counterfactual (without HPP) scenarios. Estimates are reported by country, region, and HPP class. HPP implementation was associated with a 25.2% [95% CI: 19.8% to 31.9%] reduction in excess deaths attributable to extreme heat, corresponding to 1.8 [95% CI: 1.3–2.4] avoided deaths annually per 100 000 inhabitants. This equates to an estimated 14 551 [95% CI: 10 118–19 072] total deaths avoided across all study locations following HPP implementation. No significant differences in HPP effectiveness were observed by European region or HPP class. Our findings provide robust evidence that HPPs substantially reduce heat-related mortality across Europe, accounting for temporal changes and geographical differences in risks. These results emphasise the importance of monitoring and evaluating HPPs to enhance adaptation to a warming climate.

## 1. Introduction

Europe is the fastest warming continent and has experienced an increase in extreme weather phenomena in the past two decades (EEA 2024). Intense heat waves occurred in South-Western Europe in 2003, in Eastern Europe and Russia in 2010, and across the whole continent in 2022 (Barriopedro *et al* 2011, Lhotka and Kyselý 2022, Ballester *et al* 2023, EEA-European Environment Agency 2024). Such events are likely to become a regular phenomenon in mid-latitudes by the end of the 21st century (CCAG 2024), and pose a high risk to human health.

Heat prevention plans (HPPs), including policies such as heat early warning systems and heat-health action plans, are universally considered key public health interventions to reduce health impacts of heat (Toloo *et al* 2013, McGregor *et al* 2015, Casanueva *et al* 2019). While widespread and adopted in many countries, evidence on the effectiveness of HPPs in reducing heat-related health risks is still limited. So far, most studies analysed the effect of HPP implementation in a single location by a simple comparison of the heat-related mortality risk before and after the HPP implementation (Dwyer *et al* 2022). Only a few studies investigated beneficial effects of HPPs in multiple locations with inconsistent findings across cities and regions (De’ Donato *et al* 2015, Martínez-Solanas and Basagaña 2019, Ragettli *et al* 2024). For example, a study comparing the temperature-mortality relationships in nine European cities before and after the major 2003 heatwave revealed reduced heat-related mortality risk in Rome and Paris, which implemented HPPs after 2003, but also in Athens, which did not (De’ Donato *et al* 2015). In contrast, other cities that implemented HPPs after 2003 (e.g. Budapest, Valencia, London) did not see any significant reductions in heat-related mortality. Similarly, two studies from the US received inconclusive results in the beneficial effects of heat alerts issued by the US National Weather Service (Weinberger *et al* 2017, 2021).

A factor complicating the evaluation of HPP beneficial effects is the temporal and geographical variation in mortality risks due to the underlying changes in vulnerability (Boeckmann and Rohn 2014) and uneven occurrence of major heat wave events across the continent (Lhotka and Kyselý 2022). Trends in vulnerability may result from demographic changes, other policy interventions or autonomous adaptation to a changing climate. Previous studies have identified a long-term weakening of heat-attributable mortality risk in the past century (Sheridan and Allen 2018), despite warming trends in most regions of the world. However, the declining trend in the heat-attributable risk has slowed down or even reversed in most recent years, in spite of ongoing socioeconomic development and increasing implementation of HPPs (Pascal *et al* 2021, Urban *et al* 2022). In addition, there is limited knowledge of the comparative effectiveness of HPPs in different locations and with different level of development (Ebi 2019, Dwyer *et al* 2022). So far, only one study investigated the reduction effect of HPPs with accounting for the level of their development, revealing stronger mortality reductions in provinces with more advanced HPPs, though some regions with robust plans still reported increases in heat-attributable mortality (Martínez-Solanas and Basagaña 2019).

Despite using state-of-the-art methodology such as quasi-Poisson time series regression or difference-in-differences quasi experimental study design (Dwyer *et al* 2022), the main limitation of most studies is that they did not consider these underlying trends in heat-related mortality risks. The aim of this study is to provide a comprehensive assessment of the ability of HPPs to prevent heat-related mortality across Europe. The study benefits from the partnership between two consortia, the Multi-Country Multi-City (MCC) Collaborative Research Network and the COST Action PROCLIAS, allowing the collection of a large database including daily time series data for temperature and mortality as well as a detailed classification of HPPs in selected countries. The analysis is based on state-of-the-art epidemiological methods, with the application of a three-stage analysis using a multilevel and longitudinal design that allows separating effects of HPPs from underlying regional variations in mortality risks. This setting allows us to take into account a wide array of climatic conditions and long periods after the implementation of the HPPs.

## 2. Data and method

### 2.1. Mortality and temperature data

**Figure 1.**
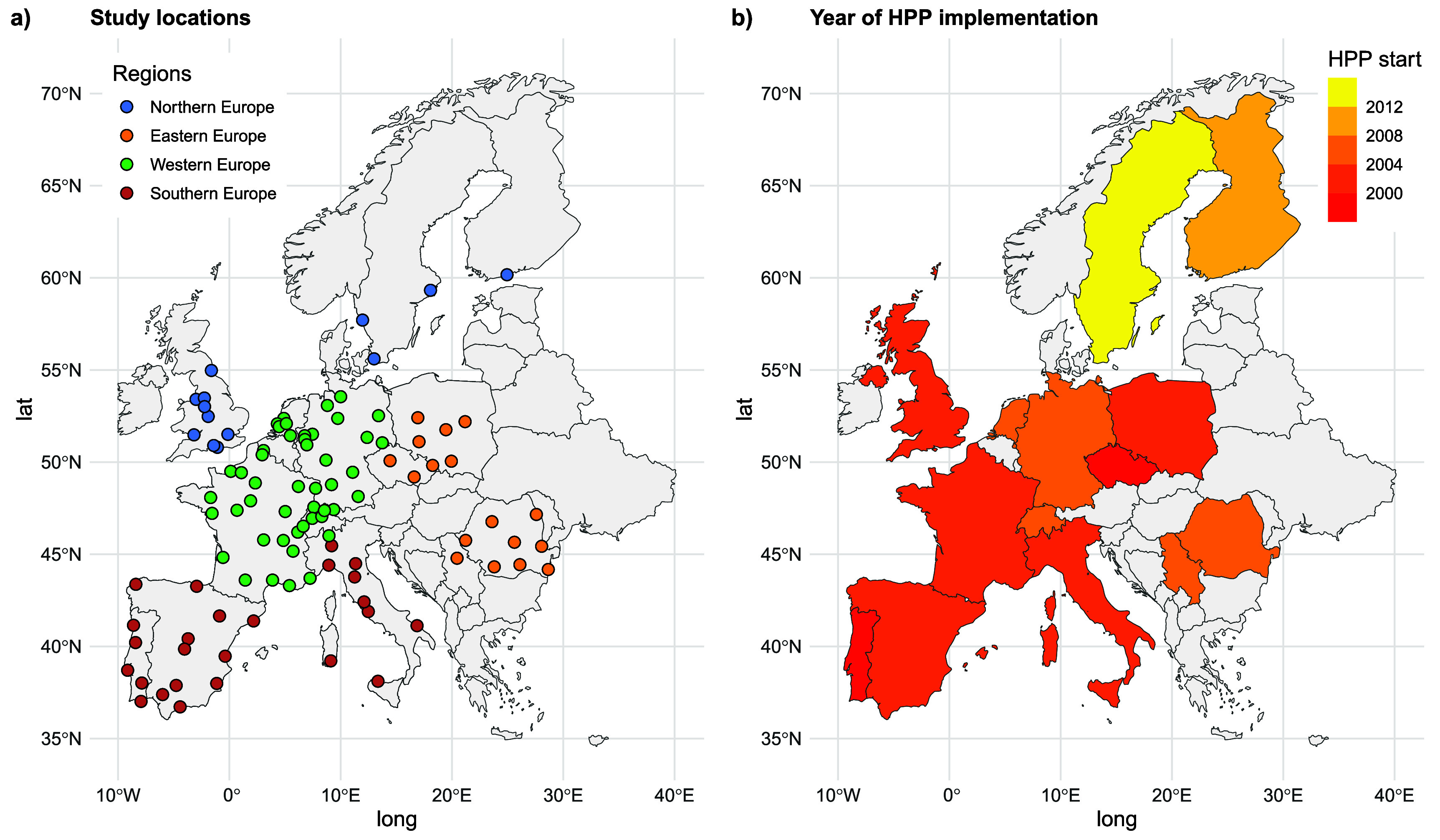
(a) Cities included in the study, (b) year of heat prevention plan implementation (HPP start) in each country.

We obtained daily death counts and daily mean temperature data in European locations available via the MCC Network (http://mccstudy.lshtm.ac.uk/), an international collaboration of research teams producing epidemiological evidence on the association between environmental stressors, climate change, and health across the globe (Gasparrini *et al* 2024). For Europe, the MCC Network has gathered data from ca. 280 locations in 19 countries. The current analysis was restricted to 102 locations in 14 European countries divided into four regions (i.e. four United Nations’ M49 geographical subregions) with available data on HPPs implementation (figure 1(a)). Differently from the M49 classification, Belgrade, Serbia, was considered Eastern Europe for the purpose of this study due to its socioeconomic and climatological proximity to this region. Mortality data spanned the period from 1990 to 2019, whilst the exact timespans differed for individual countries (figure S1, in supplementary material). For each location, mortality is represented by daily counts for all causes, or for non-external causes (International Classification of Diseases (ICD-10): A0–R99). Daily mean temperature data from weather stations representative of the selected cities were collected by MCC partners from the respective national weather services (table S1).

#### 2.2. HPPs

To assess the effectiveness of national policies to reduce heat-related mortality across Europe, we developed a catalogue of HPPs as part of the COST Action PROCLIAS in collaboration with national experts. The process relied on information collected in previously published studies (Casanueva *et al* 2019, Vanderplanken *et al* 2020, Dwyer *et al* 2022) as well as national documents available through the online portals Climate-ADAPT and Global Heat Health Information Network. The catalogue provides information for 34 European countries or regions, which were matched with the MCC locations (figure S2).

A HPP score was produced building upon the WMO/WHO’s Guidance on warning-system development (table S2) (McGregor *et al* 2015) and the methodology proposed by (Martínez-Solanas and Basagaña 2019). Briefly, we defined a total score as the number of actions implemented within 8 core elements, assigning for each action included in the HPP points between 0 and 2 depending on their importance in preventing heat-related deaths. These weights were based on an expert elicitation event (https://proclias.eu/news/elicitation-ws-prague-23), where 22 international experts from the field of atmospheric sciences and/or public health evaluated the importance of individual actions. More details on the HPP score development is provided in Section S2 in the Supplementary Material.

### 2.3. Statistical analysis

All statistical analysis was conducted in the R software (version 4.4.0), using packages *dlnm, mixmeta*, and *lmtest*. The analysis was conducted using a three-stage multilevel longitudinal design to control for spatial and temporal confounding (Sera *et al* 2019, 2020). Each stage is described separately below.

*Stage 1*

First, we used a distributed lag nonlinear model (DLNM) with quasi-Poisson parametrisation (Gasparrini 2014), to estimate the temperature-mortality association in the five warmest months of the year (May–September, typically the season when HPPs are activated) for each location. The regression was fitted separately in three-year subperiods (from 1990–1992–2017–2019, figure S1). Daily mean temperature was chosen as the exposure variable, consistently with previous large-scale assessments (Masselot *et al* 2023). The model specification and parametrisation were based on previous studies focusing on the warm season (Gasparrini *et al* 2015, Sera *et al* 2020). Briefly, an interaction term between a natural cubic spline with four degrees of freedom for day of the season and year indicator was used to control for inter- and intra-seasonal variations in mortality data. In addition, we included a categorical variable for day of the week to control for a weekly cycle. The temperature-mortality relationship was modelled with a cross-basis function, using a quadratic B-spline with two internal knots placed at the 50th and 90th percentile of the full-period location-specific temperature distribution and a natural cubic spline with two internal knots equally distributed on a logarithmic scale over 10 d of lags to control for the lagged effects of heat and a short-term mortality displacement. The overall exposure-response function was then reduced into a one-dimensional overall cumulative exposure-response curve, which expresses the location-specific relative risk (RR) of mortality compared to the subperiod- and location-specific minimum mortality temperature (MMT; Tobías *et al* 2021).

*Stage 2*

In the second stage, coefficients representing the reduced subperiod- and location-specific exposure-response functions were pooled in a multivariate mixed-effect meta-regression model with a multilevel longitudinal structure (Sera *et al* 2019). Six versions of the second-stage meta-regression model were compared to test specific aspects of heat-mortality associations and the effect of HPP implementation. Details of the models and their comparison are described in Section S3. The main model (Model 3) included i) an indicator for the presence of ${\text{HPP}}$, defined as 0 if its implementation occurred earlier or in the first year of the subperiod in which the HPP started, and 1 otherwise, and ii) an interaction term between ${\text{Region}}$ (one of the four regions defined in figure 1) and (a) linear term (${\text{Time}}$) for the middle of the subperiod, to control for region-specific long-term changes in heat-related mortality risks. The model included also random effects for intercept and time at city level, allowing differential baseline and trend in heat-related risk. Model 3 was further extended by using interaction terms with the ${\text{HPP}}$ indicator to investigate differential effects of HPP implementation by ${\text{Region}}$ (Model 4) and HPP score (Model 5), with the latter stratified in three groups (${\text{HPPclass}}$). The estimation was carried out using a maximum-likelihood estimator and a diagonal structure of the random-effect (co)variance matrix. Statistical significance of the fixed-effect interactions was tested by a likelihood ratio test (LRT) (Sera *et al* 2019).

*Stage 3*

In the third stage, we derived best linear unbiased predictions (BLUPs) from the meta-regression model to obtain improved location-specific estimates of exposure-response functions for each subperiod (Sera *et al* 2019). BLUPs were estimated by adding the residual random-effect part to fixed-effect predictions from the second-stage model, allowing them to be defined for two scenarios—*factual* and *counterfactual*. In the *factual* scenario, the HPP indicator corresponded to the observed pattern, while in the *counterfactual* scenario, the HPP indicator was set to 0 for all the subperiods. We then calculated the heat-attributable fraction of deaths (HAF) (Gasparrini and Leone 2014) in each location and each subperiod of the post-implementation period under the two scenarios. Since most HPPs have been designed to protect the population from extreme heat, we computed the HAF as the temperature-related percentage of deaths on days hotter than the 95th percentile of the location-specific summer temperature distribution over the whole study period. Finally, HAFs were used to calculate annual heat-attributable death rates per 100 000 inhabitants (HADs), using population estimates in each location for 2015 from EUROSTAT.

Eventually, the changes in HAFs (relative differences) and HADs (differences) between the counterfactual and factual scenarios in the post-implementation period were calculated to quantify the protective effect of HPP implementation in terms of the proportion of deaths avoided by HPP implementation in each country. To assess the impact of the 2003 heat wave on the results, we conducted the main analysis (Model 3) excluding data from the summer of 2003 as a sensitivity analysis.

The uncertainty of the HAF and HAD estimates, along with their changes, was quantified by generating 1000 samples of the BLUP coefficients using Monte Carlo simulations. The samples were generated assuming a multivariate normal distribution of coefficients of the second-stage meta-regression model (Masselot *et al* 2023). From these simulations, 95% empirical confidence intervals (CIs) were derived based on the empirical distribution of the coefficients.

## 3. Results

Out of 34 countries and regions listed in the HPP catalogue, data from 14 countries that provided suitable mortality as well as HPP data were included in the final analysis. The HPP scores based on the expert elicitation are shown in figure S2, with the UK and Portugal scoring the highest, having implemented actions across all core elements. Scandinavia (Sweden and Finland) and central-Eastern Europe (Czechia and Poland), on the other hand, scored the lowest due to having heat alert systems with no follow-up actions (figure S3(a)). For further assessment, the HPPs were stratified in three levels (HPP class 1–3), defined as terciles of the maximum HPP score (table S3, figure S3(b)).

The year of implementation was the key element for analysing the impact of HPPs on heat-related mortality (figure 1(b)). Figure S1 shows that the most advanced HPPs (HPP class 3) were generally implemented in Southern and Western Europe after the 2003 heat wave, while the HPPs in Northern Europe (HPP class 1) were developed later as a response to the 2010 Eastern European heat wave. In general, less developed HPPs were found in Eastern and Northern European countries compared to Southern and Western Europe.

### 3.1. Trends in heat-related mortality

**Figure 2.**
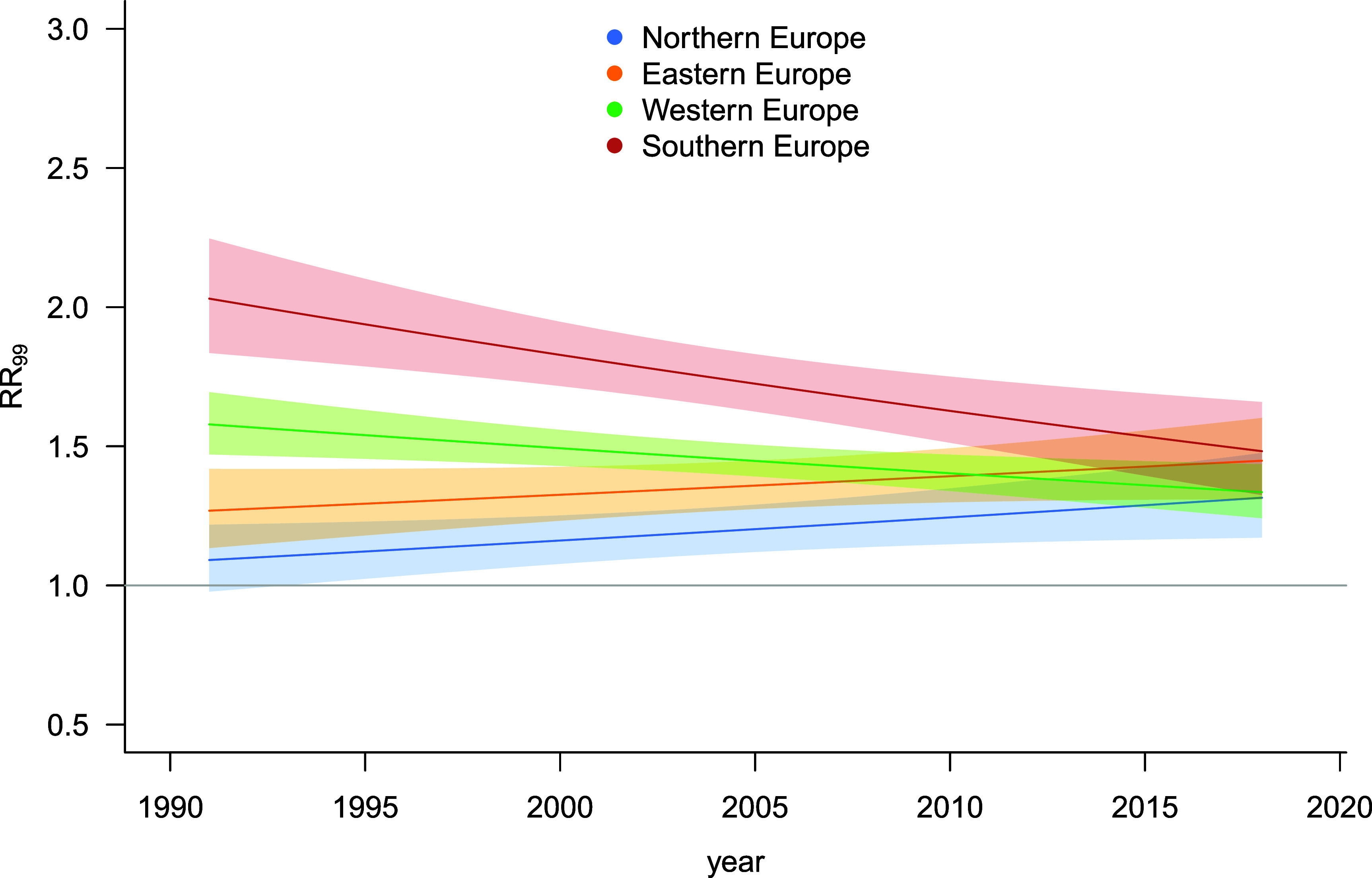
Trends in the overall relative risk of temperature-related mortality at the 99th percentile of May–September daily mean temperature distribution (RR_99_) in the four regions considered in the study (figure 1(a)).

The comparison of the various specifications of the meta-analytical model indicates strong evidence for differential trends across regions (table S4). Therefore, the results from Model 3 are reported here. Figure 2 shows trends in the overall RR of temperature-related mortality at the 99th percentile of temperature distribution compared to the subperiod- and region-specific MMT (RR_99_). The results indicate decreasing trends in RRs in populations of Southern (change in RR_99_ from 2.03 [95% CI: 1.84–2.25] in the first subperiod (1990–1992) to 1.48 [95% CI: 1.32–1.66] in the last one (2017–2019)) and Western Europe (1.58 [95% CI: 1.47–1.69] to 1.34 [95% CI: 1.24–1.44]). On the contrary, increasing trends in heat-related risks were observed in Northern (1.09 [95% CI: 0.98–1.22] to 1.32 [95% CI: 1.17–1.48]) and Eastern Europe (1.27 [95% CI: 1.13–1.42] to 1.45 [1.31–1.60]).

**Figure 3.**
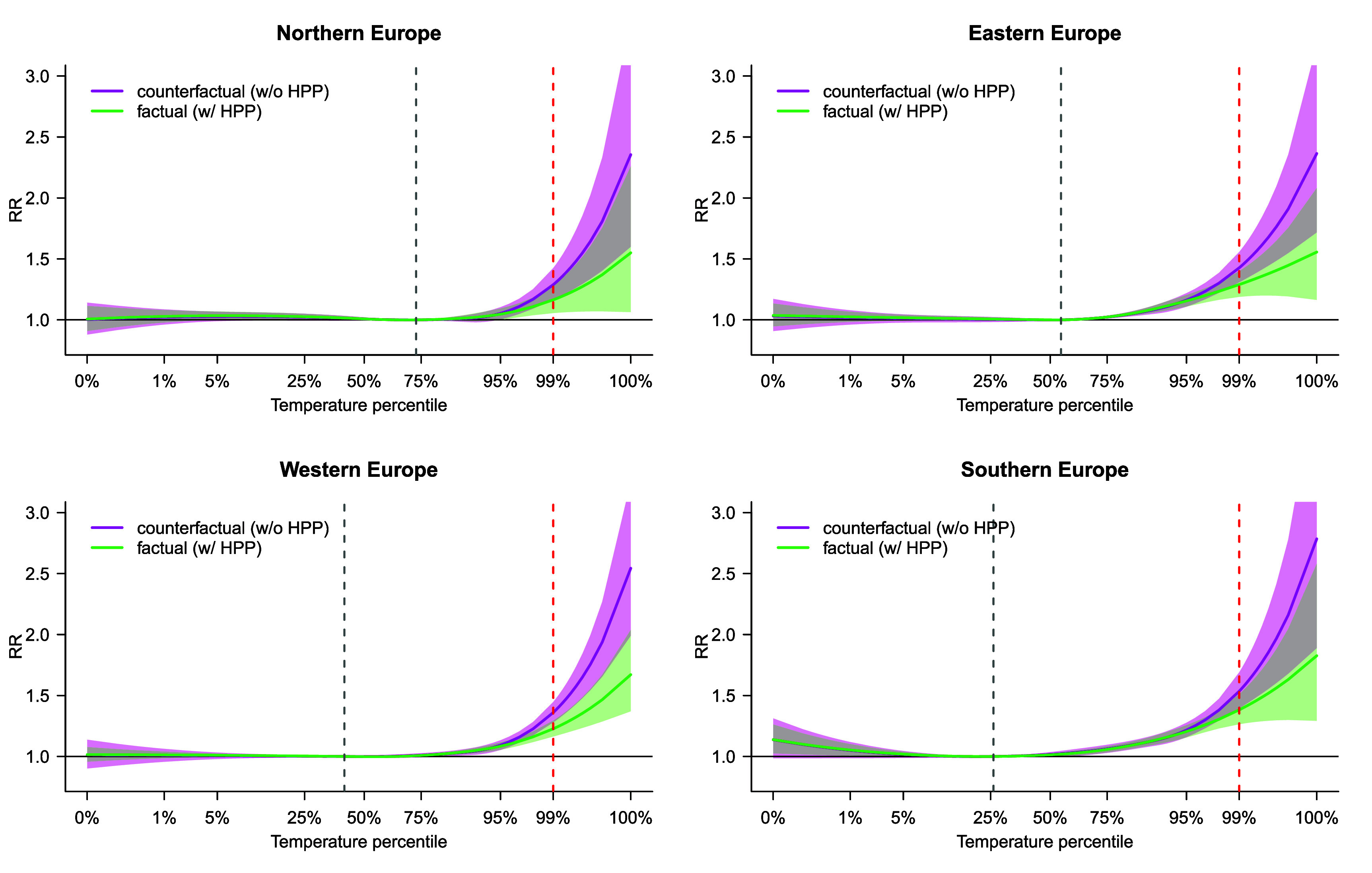
Pooled exposure-response functions (ERF) of relative risk (RR) of temperature-related mortality across the four regions under study, predicted in subperiod 2014–2016 for scenarios without (counterfactual) vs. with (factual) HPP being implemented. The *x*-axis represents relative temperatures in percentiles of May–September daily mean temperature distribution, but rescaled using the average distribution of absolute temperature across all locations. Vertical broken lines indicate the percentiles of minimum mortality temperature (grey) and 99th (red) percentiles of daily mean temperature distribution.

Figure 3 presents the difference in the pooled estimates of overall ERFs for the four regions predicted in 2015 under the two scenarios, namely without (*counterfactual*) and with (*factual*) HPP being implemented. These estimates are adjusted for regional temporal trends presented in figure 2, in addition to city-level deviations. Model 3 revealed strong evidence of an overall protective effect of HPP implementation according to LRT (*p* < 0.001) (table S4). On average, HPP implementation was associated with a reduction of RR_99_ from 1.41 [95% CI: 1.35–1.46] in the *counterfactual* to 1.27 [95% CI: 1.22–1.31] in the *factual* scenario (table S5). While the differences in RR_99_ were similar in all regions (in line with Model 3 settings), a comparison of the 95% CIs indicates that the reduction was most evident in Western Europe with RR_99_ = 1.36 [95% CI: 1.28–1.45] for the *counterfactual* and 1.23 [95% CI: 1.16; 1.29] for the *factual* scenario.

The analysis of differential effects of HPPs across regions (Model 4) seemed to suggest a larger decrease in the heat-related mortality risk in Southern Europe, with RR_99_ of 1.61 [95% CI: 1.41–1.83] vs 1.39 [95% CI: 1.27–1.52] for the *counterfactual* and *factual* scenarios, respectively, compared to Eastern Europe (RR of 1.37 [95% CI: 1.23–1.53] vs 1.29 [95% CI: 1.19–1.40]). However, LRT showed no statistical evidence (*p* = 0.44) of a differential effect of HPP based on ${\text{Region}}$ (table S4).

Similarly, Model 5 revealed limited evidence (LRT *p* = 0.19) of differences in HPP implementation between the HPP classes (table S4). Indeed, while the model confirms the protective effect of any HPP implementation, small differences were found between the types of HPPs (table S6). Given the limited evidence of differential effects of ${\text{Region}}$ and ${\text{HPPclass}}$ in Models 4 and 5, Model 3 was used further to provide estimates of the changes in heat-attributable excess mortality with HPP implementation.

### 3.2. Effects of HPP implementation on heat-attributable excess mortality

**Figure 4.**
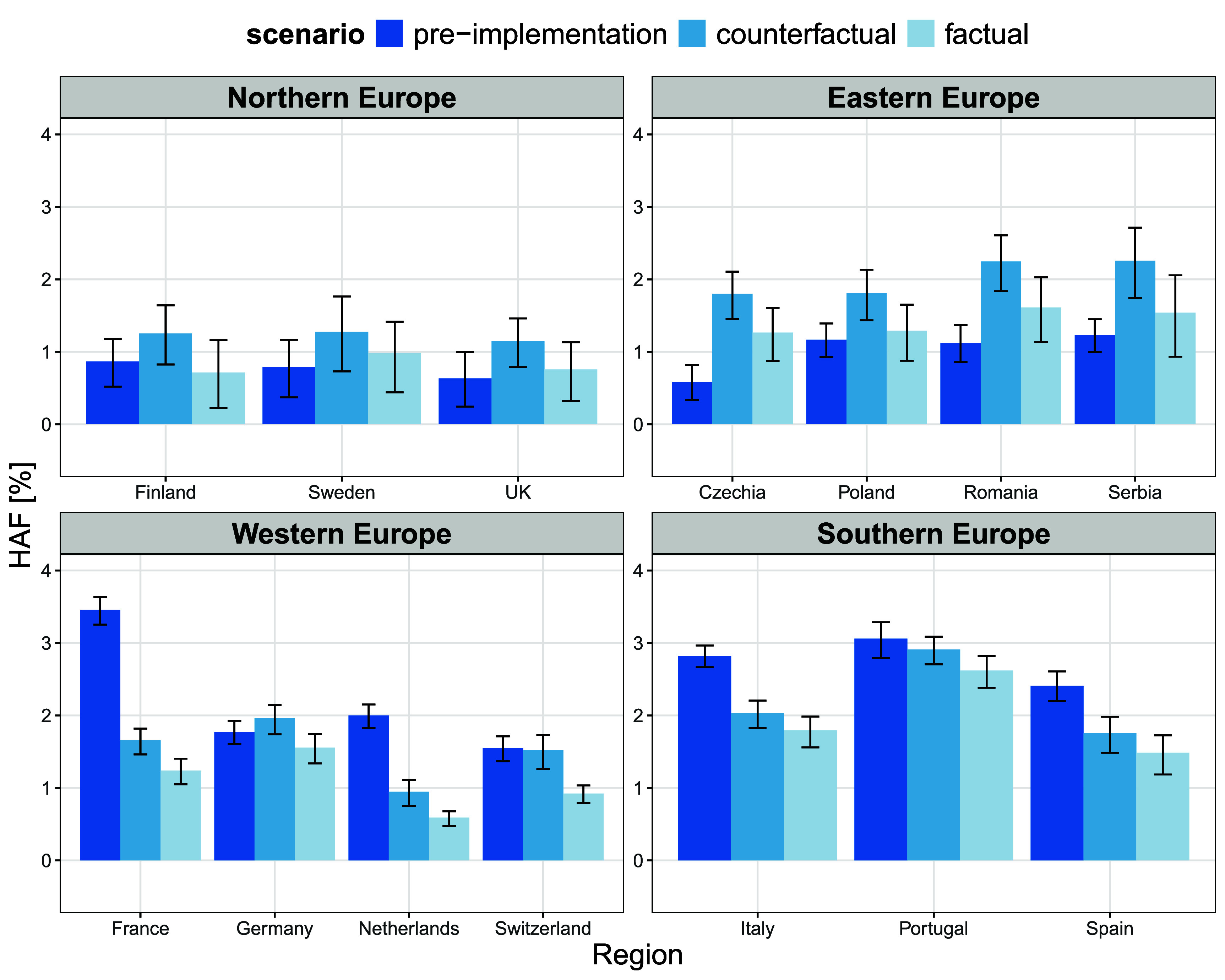
Heat-attributable fraction (HAF in %) of total May–September deaths per country before HPP implementation (pre-implementation), after HPP implementation, but without the effect of HPPs (counterfactual), and after HPP implementation with the effect of HPPs (factual). Error bars indicate 95% empirical confidence intervals calculated by Monte-Carlo simulations.

**Figure 5.**
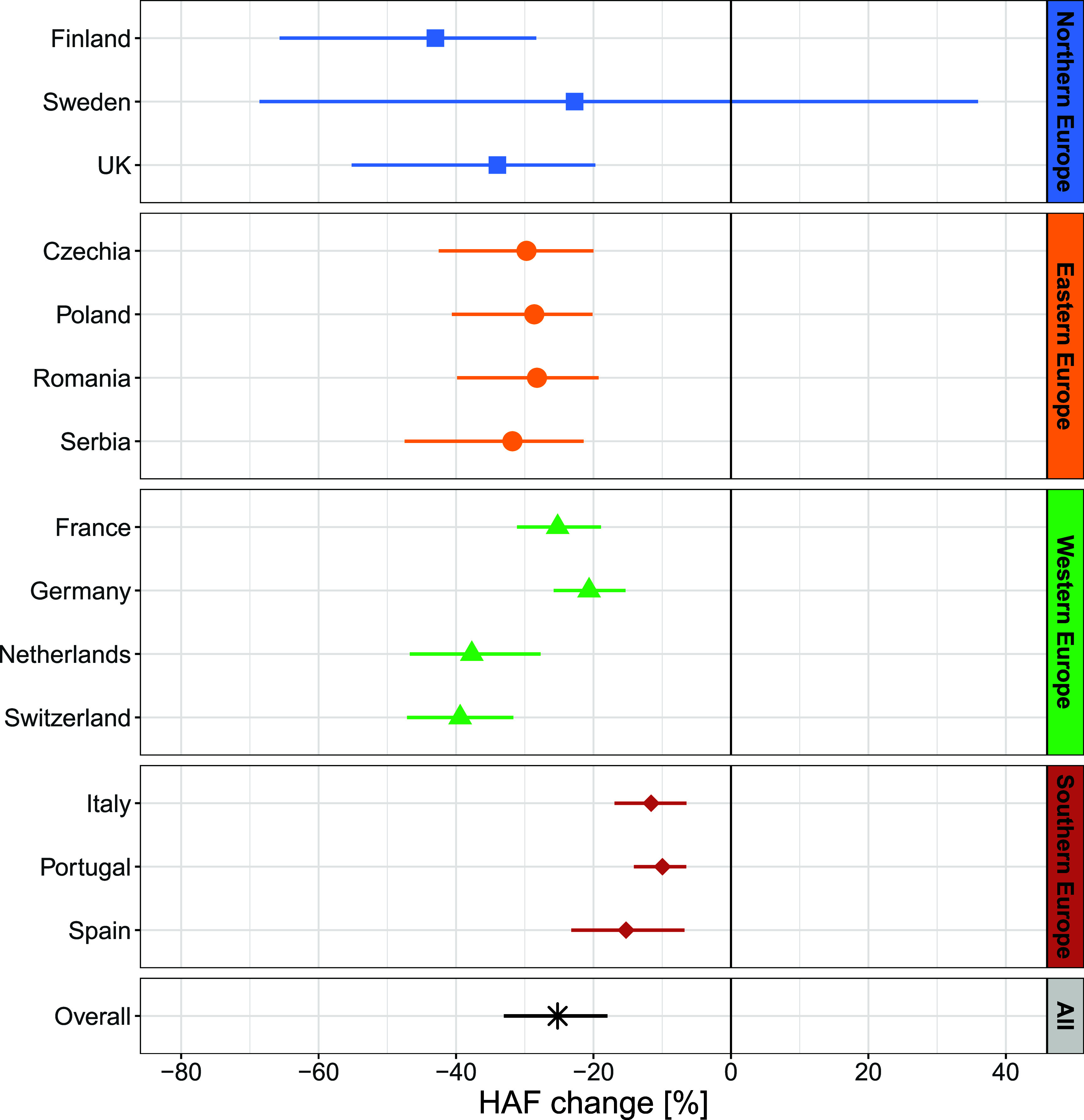
Change in heat-attributable mortality fraction (quantified as a relative difference between the *counterfactual* and the *factual* scenarios) per country associated with HPP implementation. Error bars indicate 95% empirical confidence intervals calculated by Monte-Carlo simulations (see table 1).

Figure 4 shows variations in the mean HAF in individual countries in the period before HPP implementation (pre-implementation), compared to the period after HPP implementation under both the counterfactual and factual scenarios (see figure S4 for detailed temporal changes in HAFs over individual subperiods). Across all the locations considered in this study, implementation of HPPs was associated with a 25.2% [95% CI: 19.8–31.9%] decrease in HAF compared to the counterfactual scenario (table 1, figure 5), which was equivalent to 1.8 [95% CI: 1.3–2.4] annual deaths per 100 000 inhabitants avoided due to HPPs. This implies that overall, 14 551 [95% CI: 10 118–19 072] deaths were prevented in locations of the study thanks to the implementation of HPPs.

**Table 1.** Relative differences in heat-attributable mortality fractions (HAF change) and absolute differences in heat-attributable deaths per 100 000 inh. (HAD difference) between the *counterfactual* and the *factual* scenarios in the study countries, regions and HPP classes. The values indicate the percentage of deaths and annual deaths per 100 000 inh., respectively, avoided by implementation of HPPs. The values are based on effect estimates of Model 3. Numbers in the brackets indicate 95% empirical confidence intervals estimated by Monte Carlo simulations.

Region	Country	HAF change (%)	HAD difference (annual deaths per 100 000 inh.)
Northern Europe	Finland	−43.0 (−66.6; −28.3)	−2.6 (−3.4; −1.9)
	Sweden	−22.8 (−68.6; 30.8)	−1.1 (−4.9; 2.1)
	UK	−34.0 (−55.8; −19.5)	−1.1 (−1.6; −0.6)

Eastern Europe	Czechia	−29.8 (−42.5; −20.6)	−2.3 (−2.9; −1.7)
	Poland	−28.6 (−41.3; −20.0)	−2.1 (−2.7; −1.5)
	Romania	−28.2 (−39.9; −19.8)	−2.2 (−2.8; −1.6)
	Serbia	−31.8 (−47.4; −21.5)	−4.3 (−5.5; −3.3)

Western Europe	France	−25.2 (−31.5; −19.1)	−1.4 (−1.7; −1.0)
	Germany	−20.6 (−25.7; −15.8)	−1.8 (−2.3; −1.4)
	Netherlands	−37.7 (−45.4; −29.6)	−1.1 (−1.5; −0.7)
	Switzerland	−39.4 (−47.2; −31.5)	−2.6 (−3.4; −1.9)

Southern Europe	Italy	−11.6 (−16.4; −6.7)	−0.9 (−1.2; −0.5)
	Portugal	−10.0 (−13.3; −6.7)	−1.2 (−1.6; −0.8)
	Spain	−15.3 (−23.0; −7.2)	−0.8 (−1.2; −0.4)

Mean per region	Northern Europe	−33.2 (−52.5; −20.7)	−1.6 (−2.9; −0.5)
	Eastern Europe	−29.6 (−42.8; −20.7)	−2.8 (−3.5; −2.0)
	Western Europe	−29.2 (−34.8; −23.7)	−1.7 (−2.2; −1.3)
	Southern Europe	−11.9 (−16.6; −6.9)	−1.0 (−1.3; −0.6)

Overall mean		−25.2 (−31.9; −19.8)	−1.8 (−2.4; −1.3)

Northern European countries were those with the largest impact of HPP on HAFs, with an average decrease in HAF of 33.2% [95% CI: 20.6–52.5%], while Southern Europe revealed the smallest reduction (HAF decrease of 11.9% [6.9–16.6%], table 1). However, the range of 95% CIs suggests a larger uncertainty of the estimates for Northern and Eastern Europe, likely related to the smaller number of locations considered in these regions (figure 5). This was true especially for Sweden with HAF decrease by 22.8% [95% CI: −30.8 to 68.6%] (an equivalent to annual 1.1 [95% CI: −2.1–4.9] avoided deaths per 100 000 inhabitants). In the UK, on the other hand, we observed the fourth largest and strong effect of HPP implementation according to mean HAF decrease (by 34.0% [95% CI: 19.5–55.8%]), although the change in HAD was the same as in Sweden (1.1 [95% CI: 0.6–1.6]). A sensitivity analysis revealed that even after excluding the summer of 2003 from the analysis, the implementation of HPPs was associated with a weaker, but still evident reduction of heat-attributable mortality by 15.2% [95% CI: 4.1–23.7%] across all locations (figure S5).

## 4. Discussion

This study investigated the impact of HPP implementation on heat-related mortality across 102 locations in 14 European countries over 30 years. Results show that HPPs were associated with a 25% reduction in excess deaths attributable to extreme heat (HAF) compared to the counterfactual scenario without HPPs. This estimate accounted for differential temporal variations in heat-related mortality risks observed in the four European regions. Yet, counter to expectations, we found little evidence that the protective effect of HPPs varied systematically by region or by HPP class.

### 4.1. Temporal changes in heat-related mortality

Studies have documented declining trends in heat-related mortality risk during past decades, attributed to positive socioeconomic development and healthcare improvements (Boeckmann and Rohn 2014, Sheridan and Allen 2018). For instance, a global study of 305 locations in 10 developed countries revealed consistent reductions in heat-related mortality in all regions, except for Australia and Brazil (Vicedo-Cabrera *et al* 2018). However, more recent data suggest abatement of the declining trends or even increased heat-related mortality risks (Pascal *et al* 2021, Urban *et al* 2022, Ballester *et al* 2023). Our results also reflect this trend, especially in locations where the most recent mortality data were available (i.e. for the subperiod 2017–2019). This suggests that accelerated trends in increasing frequency and intensity of heatwaves may challenge heat prevention and adaptation measures in the whole of Europe (Pascal *et al* 2021, Miranda *et al* 2023).

### 4.2. The effect of HPP implementation

Previous research suggested that HPPs may reduce adverse health outcomes associated with heat, although in some cases using indirect evidence and with inconsistent findings. The epidemiological literature suggests that other factors, such as climate variability, demographic changes, physiological adaptation, healthcare system improvements, and general socio-economic development (Boeckmann and Rohn 2014), play an important role and can generate temporal trends in heat vulnerability. Despite the recognised importance of these factors, most previous assessments of the effectiveness of HPPs have not adjusted for underlying trends in heat vulnerability, nor have they considered temporal variations in temperature distribution. The longitudinal meta-regression used in this study enabled us to adjust the impact of HPP implementation for both overall trends in heat-related mortality related to long-term changes in mortality data (e.g. population aging) and spatio-temporal variations in temperature distribution (e.g. occurrence of major heat wave events), and to reveal a beneficial effect of HPP implementation even in countries with increasing heat vulnerability.

### 4.3. The role of HPP class

To our knowledge, no study to date has provided a comprehensive analysis of the beneficial effect of HPPs across Europe, taking into account different characteristics of HPPs in individual countries and separating the effect of HPP implementation from the underlying trends. A nation-wide study in Spain found stronger mortality reductions in provinces with more advanced HPPs, though some regions with robust plans still reported increases in heat-attributable mortality (Martínez-Solanas and Basagaña 2019). Our results revealed limited evidence that more developed HPPs result in a stronger protective effect. This may reflect large uncertainties associated with the HPP data collection and evaluation. In each country, the effective operation of individual HPP elements is subject to actual implementation at the regional and local scale, which is influenced by various factors, such as the amount of financial and human resources provided for HPP implementation, the level of harmonization between national and regional action plans, or the level of involvement of local health and social institutions (Martinez *et al* 2022). Although several studies evaluated the status of HPPs in Europe (Casanueva *et al* 2019, Vanderplanken *et al* 2020, Dwyer *et al* 2022), none of them were able to collect information fully representative for the whole of Europe and all regional levels due to difficulties with data collection (Martinez *et al* 2022). Similarly, in this study we were not able to compare the HPPs at the regional and local level and verify if the actions declared at the national level were really implemented in each location. Therefore, the national HPP score may not fully reflect the actual situation in each location.

Moreover, different political and socio-economic conditions may influence the effectiveness of similar HPP elements across countries, leading to variability in estimated HAF decreases even among locations with comparable HPP scores. As summers similar to or even hotter than those of 2003, 2010 or 2022 are expected to become more frequent in Europe in the next decade, it is essential to intensify cross-border exchange of the good practice in development and implementation of HPPs, including a standardised evaluation of national and regional HPPs and a larger focus on long-term adaptation measures (Pascal *et al* 2021, Martinez *et al* 2022).

### 4.4. Limitations

Beside the challenges in HPP data collections, several limitations of the study need to be acknowledged. First, the analysis included data from a selection of cities in selected countries, excluding non-urban areas, that may not be fully representative of the four European regions. Second, temporal variations in heat-related mortality risks are modelled with region-specific linear terms, which can fail to capture more complex and local trends. Third, the analysis does not directly control for seasonal behavioural factors such as mass European-wide tourism during the summer season. Given the character of mortality data, which is defined by the place of residence of each deceased, it is impossible to track these patterns. In addition, the analysis does not address local environmental quality status (e.g. air pollution) as well as it did not directly account for specific drivers of heat adaptation such as air conditioning, which has been previously found as associated with a reduction in heat-related risks (Sera *et al* 2020), although it is expected that its effects are incorporated into the temporal trends.

## 5. Conclusion

This study provides the most comprehensive assessment to date of the potential of HPPs for reducing heat-related mortality in Europe. It systematically explores the association between HPP implementation and heat-related mortality, employing state-of-the-art epidemiological designs and statistical techniques that account for temporal variations and geographical differences in heat-related mortality. This robust evaluation highlights a significant association between the implementation of HPPs and a 25% reduction in heat-related mortality across 102 locations in 14 European countries.

While our findings indicate that HPPs are generally effective in reducing heat-related excess mortality, challenges include limited access to accurate information on HPPs in specific locations, inconsistent regional implementation, and difficulties in tracking HPP updates. Given the increasing frequency and intensity of heatwaves in Europe, the findings emphasise the importance of a nuanced understanding of broader determinants of heat vulnerability. Refining and unifying HPP evaluation metrics is crucial to ensure a comprehensive assessment of their effectiveness in preventing heat-related mortality across Europe.

## Data Availability

Anonymised mortality data used in this study were collected by collaborators within the MCC Network under a data−sharing agreement (http://mccstudy.lshtm.ac.uk/) and cannot be made publicly available. The exposure–response curve coefficients from the Stage 1 analysis, along with the complete R code for replicating the subsequent analyses, will be made available in the GitHub (https://github.com/urbanales/MCC−HEWS) and Zenodo (https://doi.org/10.5281/zenodo.17507283) repositories within one month of manuscript acceptance. Researchers interested in accessing the raw mortality data for the included cities may contact the corresponding author (Aleš Urban, https://urban@ufa.cas.cz) or the MCC Network coordinators.
